# Exploration of the regulatory mechanisms of regeneration, anti-oxidation, anti-aging and the immune response at the post-molt stage of *Eriocheir sinensis*

**DOI:** 10.3389/fphys.2022.948511

**Published:** 2022-09-21

**Authors:** Meiyao Wang, Jiachun Ge, Xingkong Ma, Shengyan Su, Can Tian, Jianlin Li, Fan Yu, Hongxia Li, Changyou Song, Jiancao Gao, Pao Xu, Yongkai Tang, Gangchun Xu

**Affiliations:** ^1^ Key Laboratory of Freshwater Fisheries and Germplasm Resources Utilization, Ministry of Agriculture and Rural Affairs, Freshwater Fisheries Research Center, Chinese Academy of Fishery Sciences, Wuxi, China; ^2^ Wuxi Fisheries College, Nanjing Agricultural University, Wuxi, China; ^3^ Freshwater Fisheries Research Institute of Jiangsu Province, Nanjing, China; ^4^ College of Fisheries and Life Science, Shanghai Ocean University, Shanghai, China

**Keywords:** Eriocheir sinensis, heart, regeneration, post-molt stage, comparative transcriptome

## Abstract

*Eriocheir sinensis* is widely appreciated by the surrounding population due to its culinary delicacy and rich nutrients. The *E. sinensis* breeding industry is very prosperous and molting is one of the important growth characteristics. Research on the regulation of molting in *E. sinensis* is still in the initial stages. There is currently no relevant information on the regulatory mechanisms of heart development following molting. Comparative transcriptome analysis was used to study developmental regulation mechanisms in the heart of *E. sinensis* at the post-molt and inter-molt stages. The results indicated that many regulatory pathways and genes involved in regeneration, anti-oxidation, anti-aging and the immune response were significantly upregulated after molting in *E. sinensis*. Aside from cardiac development, the differentially expressed genes (DEGs) were relevant to myocardial movement and neuronal signal transduction. DEGs were also related to the regulation of glutathione homeostasis and biological rhythms in regard to anti-oxidation and anti-aging, and to the regulation of immune cell development and the immune response. This study provides a theoretical framework for understanding the regulation of molting in *E. sinensis* and in other economically important crustaceans.

## Introduction

The Yangtze River is 6,397 km long and ranks third in the world in both length and water flow. The Yangtze River basin has very rich biodiversity and contains 424 different fish species and more than 29,000 kinds of phytoplankton and benthic organisms, making it an important region for global biodiversity conservation (Lv et al., 2016; Jin et al., 2022; Zhang et al., 2022). *E. sinensis* is an economically valuable catadromous species found in the Yangtze River. Due to its high mobility and ability for osmotic regulation, *E. sinensis* now has a global distribution that includes Europe and America (Gillard et al., 2017; Spiridonov and Zalota, 2017). *E. sinensis* is rich in nutrients and flavor and is known as “one of the three delicacies of the Yangtze River”. It has high economic value and the *E. sinensis* breeding industry has developed rapidly (Song and Zhao, 2018; Wang et al., 2018). Molting is a typical characteristic of crustaceans including crabs and shrimps, allowing them to grow discontinuously throughout their life cycle. Study of the regulatory mechanism of *E. sinensis* molting is important for the protection of wild crab resources and for the development of an economically viable crab breeding industry.

Based on the morphological characteristics of the setae and on the retraction degree of the epidermis, the molting cycle can be divided into four stages: pre-molting (D), inter-molting (C), post-molting (AB) and molting (E). During the post-molt stage, water is quickly absorbed and the exoskeleton gradually hardens. During the inter-molt stage, the exoskeleton continues to harden and mineralize, while the muscle gradually enlarges and the water content decreases. During the pre-molt period, the old skeleton decomposes and is absorbed, while a new skeleton and pigment layer begin to form (Kang et al., 2012). Molting appears to be coordinated by several hormones produced in the central nervous and endocrine systems. This occurs mainly through secretion of the molting inhibition hormone (MIH) by the X-organ/sinus gland complex in the eye-stalk. In addition, a transcription factor composed of the ecdysone receptor and retinoid X receptor acts on Y organs to regulate the synthesis and secretion of the hormone ecdysone by these organs. These two antagonistic hormones act jointly to regulate the molting process of *E. sinensis* (Das et al., 2018).

Current research on the regulation of *E. sinensis* molting focuses mainly on the influence of external environmental factors and nutritional elements, including temperature, pH, vitamins, etc. (Yu et al., 2018; Chen et al., 2019; Wang et al., 2020a; Liu et al., 2021a; Zhang et al., 2021). Moreover, the role of several regulatory genes such as V-ATPase subunit B (VATB), transforming growth factor-beta type I receptor (TGFBR1) and S6 kinase has also been studied (Tian et al., 2019; Hou et al., 2020; Tian et al., 2020). The results indicated that V-ATPase subunit B plays essential roles in the cuticle formation process of *Eriocheir sinensis*. Transforming growth factor-β type I receptor regulates gonad and muscle development of *E. sinensis*. S6 kinase also plays an important regulatory role in muscle growth during *E. sinensis* molting process (Tian et al., 2019; Hou et al., 2020; Tian et al., 2020).

Following molting, the newly formed epidermis is soft and therefore prone to invasion and infection by pathogens. Frequent death after molting greatly influences the survival rate of adult *E. sinensis* and is one of the main problems of the *E. sinensis* culture industry (Wang et al., 2015). So far there have been few reports on the regulation of *E. sinensis* at the post-molting stage, with only one study reporting on the structure and composition of the exoskeleton during the molting process (Tian et al., 2013).

As one of the critical organs and a core component of the circulatory system, the heart plays an important role in various life activities such as development and reproduction (Goepel and Wirkner, 2020). Currently, only a few studies have been published on the heart in crabs and these involve the influence of different environmental factors such as hypoxia and temperature on the heart rate (Kushinsky et al., 2019; Zainal and Noorani, 2019; Singh et al., 2020a; Singh et al., 2020b; Levinton et al., 2020). The results indicated that crab performed cardiac compensation in response to declining dissolved oxygen. They had different strategies on heart rate under water, air and different temperature condition. Their heart rate had strong dependence to temperature. Their heart rhythm stability was better than polyric rhythm under high temperature condition (Kushinsky et al., 2019; Zainal and Noorani, 2019; Singh et al., 2020a; Singh et al., 2020b; Levinton et al., 2020). In contrast to vertebrates, the hearts of crustaceans have strong regenerative potential. After each molting, the hearts of crustaceans such as crabs and shrimps can regenerate (Xiong, 2020). It is therefore interesting to explore the regulatory mechanism of heart development in *E. sinensis* after molting. High-throughput sequencing allows molecular analysis to be carried out on a broader and more profound level (Min et al., 2019; Avarre, 2020; Liu et al., 2021b; Feng et al., 2022). Studies on the regulation of molting in *E. sinensis* are still at the preliminary stage and hence transcriptome analysis should allow rapid screening of the regulatory pathways and genes involved.

In the present study, comparative transcriptome analysis was performed on the heart tissue of *E. sinensis* at the post-molt and inter-molt stages. The aim was to identify critical regulatory pathways and genes during the post-molt developmental stage of *E. sinensis*, thereby providing a theoretical framework for better understanding of this process. This study should also provide a theoretical basis for use in the breeding industries of *E. sinensis* and other crustaceans, as well as for further research into organ regeneration in vertebrates.

## Materials and methods

### Ethics statement

The study was approved by the Animal Care and Use Committee of the Freshwater Fisheries Research Center at the Chinese Academy of Fishery Sciences. All the experiments conformed to the Guidelines for the Care and Use of Laboratory Animals set by the Animal Care and Use Committee of the Freshwater Fisheries Research Center (2003WXEP61, Jan 6th of 2003), and the study was carried out under a field permit (No. 20182AC1699).

### Experimental crabs and sample collection

One-year-old juvenile *E. sinensis* crabs (average body weight of (11.6 ± 0.68 g)) were obtained from the Jiangsu Noah’s Ark Agricultural Science and Technology Co. Ltd. Animals at the same developmental stage were selected and cultured in three aquariums. Twenty female *E. sinensis* crabs and the same number of male juveniles were grown in the same aquarium. The aquariums were continuously aerated and the water quality monitored every day, Water temperature was (19 ± 0.5)°C, pH was (7.5 ± 0.2), concentration of dissolved oxygen was (6 ± 0.3) mg/L, concentration of NH_3_-N and NO_2_^−^ was lower than 0.1 mg/L and 0.005 mg/L, respectively. *E. sinensis* were given a compound feed each day at 14:00 and 17:00. The molting stage was determined according to Kang et al. (Wang et al., 2018). Cameras were installed in each aquarium and the molting process was observed continuously for 24 h each day. The heart was collected within 30 min after molting, with one male sample and one female sample collected from each tank. The same number of heart samples was also collected from crabs at the inter-molt phase. The body size parameters of all *E. sinensis* crabs were measured before heart collection.

### Total RNA extraction and illumina sequencing

Total RNA was extracted with RNAiso reagent according to the manufacturer’s instructions (TaKaRa, Japan). Equal amounts of total RNA from the heart of one female crab and one male crab at the same developmental stage and from each tank were pooled to form one sample. In total, three samples were obtained from the post-molt stage (MP) and three from the inter-molt stage (MI). RNA samples were checked for quality and the quantification of extracted total RNA, construction of cDNA library, and high-throughput sequencing were performed according to the methods reported in our previous study (Wang et al., 2020b). The raw data generated in this study was submitted to the NCBI (NCBI, United States) with accession number PRJNA836628.

### Data filtering and assembly

Raw data were filtered using the NGS QC TOOLKIT V2.3.3 software (Roche, United States). Some low quality reads, contaminated reads, and primer and adapter sequences were removed (Patel and Jain, 2012). The filtered clean data was assembled using Trinity software (v2.2.0) (Grabherr et al., 2011).

### Transcriptome annotation

Unigenes were aligned in accordance with the following databases: non-redundant protein (Nr), non-redundant nucleotides (Nt), Swiss-prot (http://www.uniprot.org/downloads), clusters of orthologous groups for eukaryotic complete genomes (KOG, ftp://ftp.ncbi.nih.gov/pub/COG/KOG/kyva), and the Kyoto Encyclopedia of Genes and Genomes (KEGG, http://www.genome.jp/kegg/pathway.html) (Altschul et al., 1990; Kanehisa et al., 2008). Gene ontology (GO) homology annotation was carried out using Blast2GO software (Conesa et al., 2005).

### Differential gene expression analysis

Differential gene expression analysis was carried out using the DESeq R package (1.18.0) (Anders and Huber, 2012). Fold-change was calculated as the ratio of the expression level of genes in the MI sample to the MP sample. |log2foldchange| > 1 and *padj* < 0.05 (adjusted *p* value) were set as the cutoff thresholds for DEGs. The detailed method for DGE analysis was described in our previous study (Wang et al., 2020b). GO and KEGG enrichment analyses were carried out on DEGs (padj <0.05). Finally, the top 30 GO terms and top 30 KEGG pathways were identified using methods described in our previous study (Wang et al., 2020b).

### Quantitative real-time PCR (qPCR) validation

The accuracy of high-throughput sequencing data was validated with qPCR. Ten DEGs were randomly selected from the transcriptome data for qPCR analysis using the ABI 7500 real-time PCR system (ABI, United States). Primers were designed with Primer Premier six software and the primer sequences are shown in Supplementary Material S1. Beta-actin was used as the internal reference. Amplifications were performed with the following program: 95°C for 30 s, 40 cycles of 95°C for 5 s, 60 °C for 35 s, and 72°C for 52 s. Each sample was studied in triplicate and gene expression levels were calculated with the 2^−ΔΔCT^ method (Livak and Schmittgen, 2001).

### Statistical analysis

Statistical significance (*p* < 0.05) was calculated using one-way ANOVA and Duncan’s multiple range tests (SPSS 21.0). Values were shown as (Mean ± Standard Error). The minimum significance level was set to 0.05. When distribution of data was skewed, the Dunn–Bonferroni post hoc method following Kruskal–Wallis test was used (Cai et al., 2020a).

## Results

### Sequencing and assembly of the *E. sinensis* heart transcriptome

Body size parameters for *E. sinensis* at post-molt and inter-molt stages are shown in Table 1. In total, 271,145,476 clean data were generated (Table 2). In this study, Q20 value were more than 95%, these indicated that base calling accuracy for more than 95% data reached 99% and thus met the requirement for further analysis. After assembly, 169,812 unigenes were obtained. Of these, 103,924 unigenes ranged from 501 to 1000bp, 6,113 unigenes were grater than 1000bp in length. The average length was 961bp, N50 was 1109bp.

**TABLE 1:** Body size parameters for the *E. sinensis* study samples.

ID	Weight (g)	Carapace length (mm)	Carapace width (mm)
MP1-F	9.1	25.1	27.7
MP1-M	10.6	25.7	29.9
MP2-F	8.9	24.6	27.1
MP2-M	11.1	26.5	28.8
MP3-F	8.8	24.9	27.2
MP3-M	9.9	25.6	27.5
MI1-F	9.3	24.3	27.9
MI1-M	12.1	27.4	29.9
MI2-F	9.3	24.6	28.1
MI2-M	10.3	25.9	28.9
MI3-F	9	23.1	26.1
MI3-M	11.6	26.6	29.1

NOTE: MP1-F ∼ MP3-F: three female *E. sinensis* at post-molt stage in three aquariums; MP1-M ∼ MP3-M: three male *E. sinensis* at post-molt stage in three aquariums.

MI1-F ∼ MI3-F: three female *E. sinensis* at inter-molt stage in three aquariums; MI1-M ∼ MI3-M: three male *E. sinensis* at inter-molt stage in three aquariums.

**TABLE 2:** Summary of heart transcriptome sequencing for *E. sinensis*.

Sample	Raw reads	Raw bases	Clean reads	Clean bases	Q20 (%)	GC (%)
MP1	45,247,234	6,787,085,100	44,927,568	6,638,048,172	95.26	49.9
MP2	45,992,206	6,898,830,900	44,973,304	6,646,604,598	96.02	50.3
MP3	46,182,912	6,927,436,800	45,784,144	6,773,764,105	95.9	51.1
MI1	45,969,580	6,895,437,000	45,114,446	6,683,254,030	95.70	50.64
MI2	45,288,168	6,793,225,200	4,4501,478	6,592,448,951	95.61	50.1
MI3	46,311,928	6,946,789,200	45,844,536	6,788,658,891	95.59	49.7

NOTE: MP1-3: three replicates of heart of postmolt *E. sinensis*; MI1-3:three replicates of heart of Intermolt *E. sinensis*.

Q20: ratio of bases with Phred quality score larger than 20 in raw bases.

### Top 30 GO enrichment analysis of DEGs at the post-molt and inter-molt stages

**FIGURE 1:**
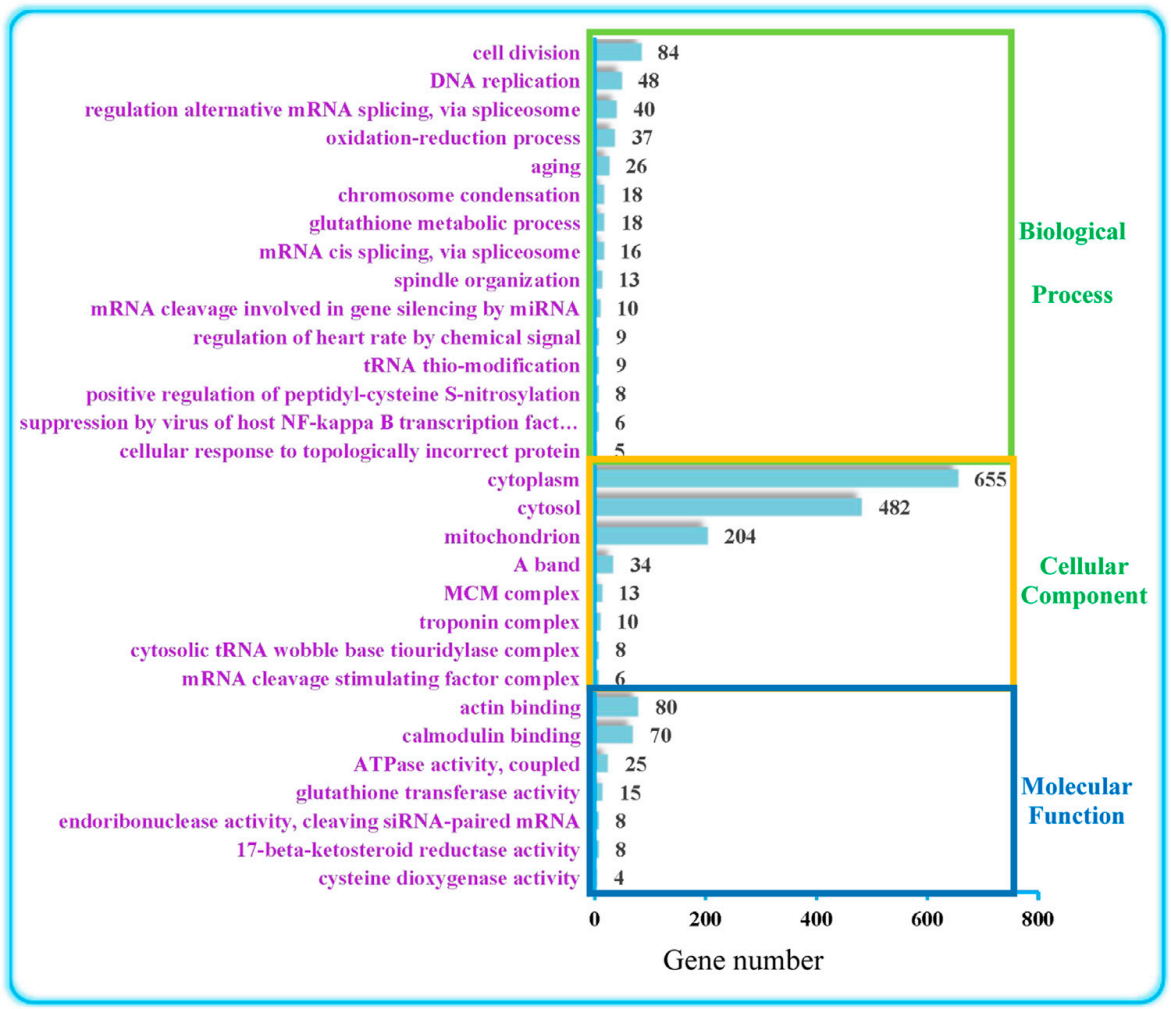
Top 30 GO terms. The numbers on the right represent the number of DEGs in each term.

As shown in Figure 1, GO can be divided into the three levels of Biological Process (BP), Molecular Function (MF), and Cellular Component (CC). These were further classified using the online program Ontobee analysis. The results indicated that BP was mainly involved in the regulation of anti-oxidation (cellular response to topologically incorrect protein, positive regulation of peptidyl-cysteine S-nitrosylation, glutathione metabolic process, aging, oxidation-reduction process), circulatory system regulation (regulation of heart rate by chemical signal) and nucleic acid metabolic processes (tRNA thio-modification, mRNA cleavage involved in gene silencing by miRNA, mRNA cis splicing via spliceosome, regulation of alternative mRNA splicing via spliceosome, DNA replication), cytoskeleton and organelle organization (spindle organization, chromosome condensation). CC mainly involved the regulation of myocardial movement (troponin complex, A band), organelle relevant to energy metabolism (mitochondrion) and organelle relevant to nucleic acid metabolism (mRNA cleavage stimulating factor complex, cytosolic tRNA wobble base tiouridylase complex). MF involved mainly binding activity relevant to cytoskeleton protein and signal transduction regulatory protein (calmodulin binding, actin binding), antioxidant enzyme regulation (cysteine dioxygenase activity, 17-beta-ketosteroid reductase activity, glutathione transferase activity), energy metabolism relevant enzyme activity (ATPase activity, coupled) and nuclease activity (endoribonuclease activity, cleaving siRNA-paired mRNA).

### Top 30 KEGG enrichment analysis

**FIGURE 2:**
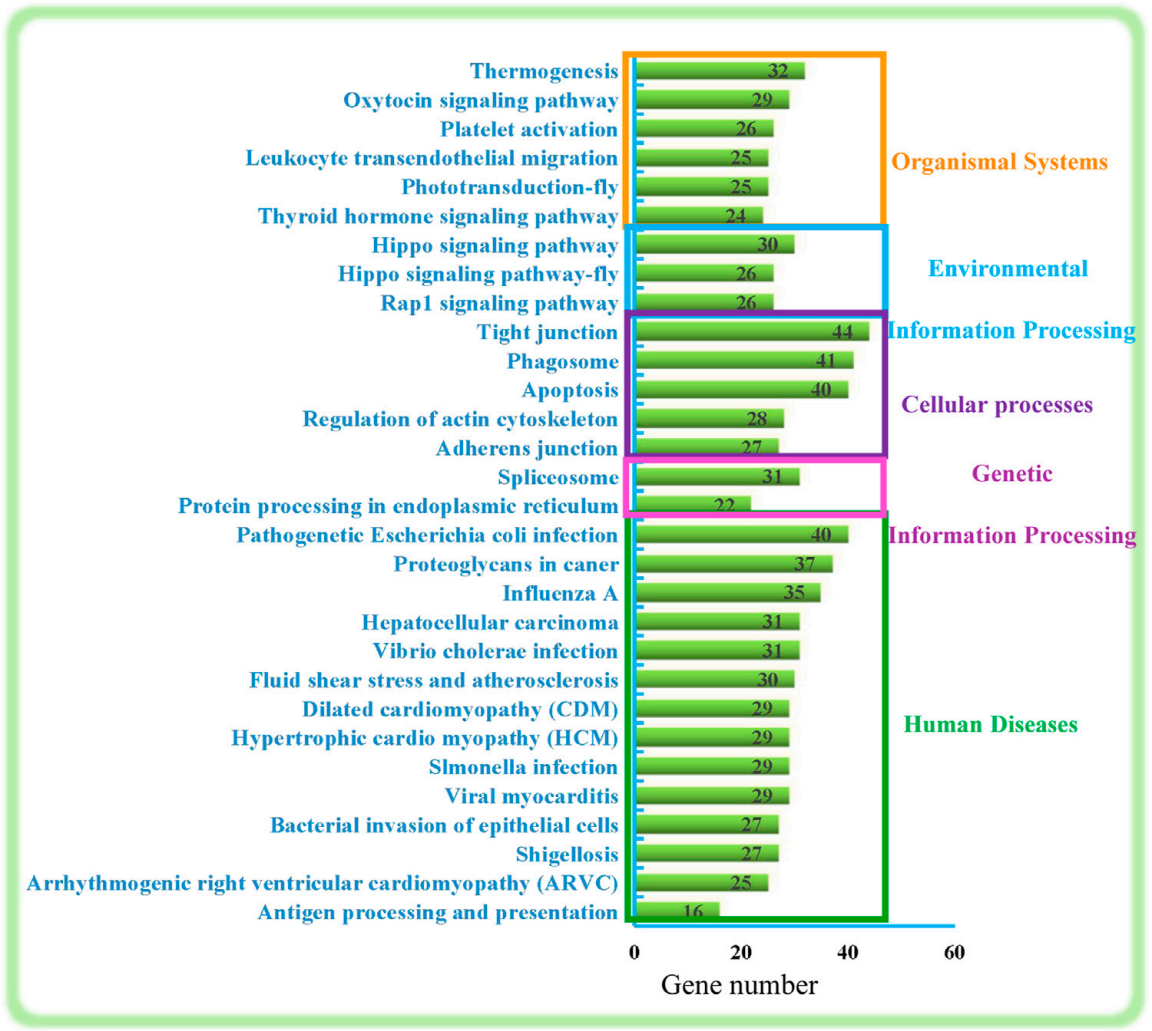
Top 30 KEGG pathways. The numbers on the right represent the number of DEGs in each term.

As shown in Figure 2, the top 30 KEGG pathways were classified into five categories: Organismal Systems, Environmental Information Processing, Cellular processes, Genetic Information Processing, and Human Diseases. In general, these mainly involve regulation of the immune system (Leukocyte transendothelial migration, the phagosome, and antigen processing and presentation), regulation of regeneration and development (Rap1 signaling pathway, Hippo signaling pathway-fly, Hippo signaling pathway, Thyroid hormone signaling pathway, regulation of actin cytoskeleton and apoptosis) and genetic information processing and signal transduction (protein processing in endoplasmic reticulum, spliceosome, adherens junction, tight junction).

### Regulatory network between post-molt and inter-molt stages in the heart of *E. sinensis*

**FIGURE 3:**
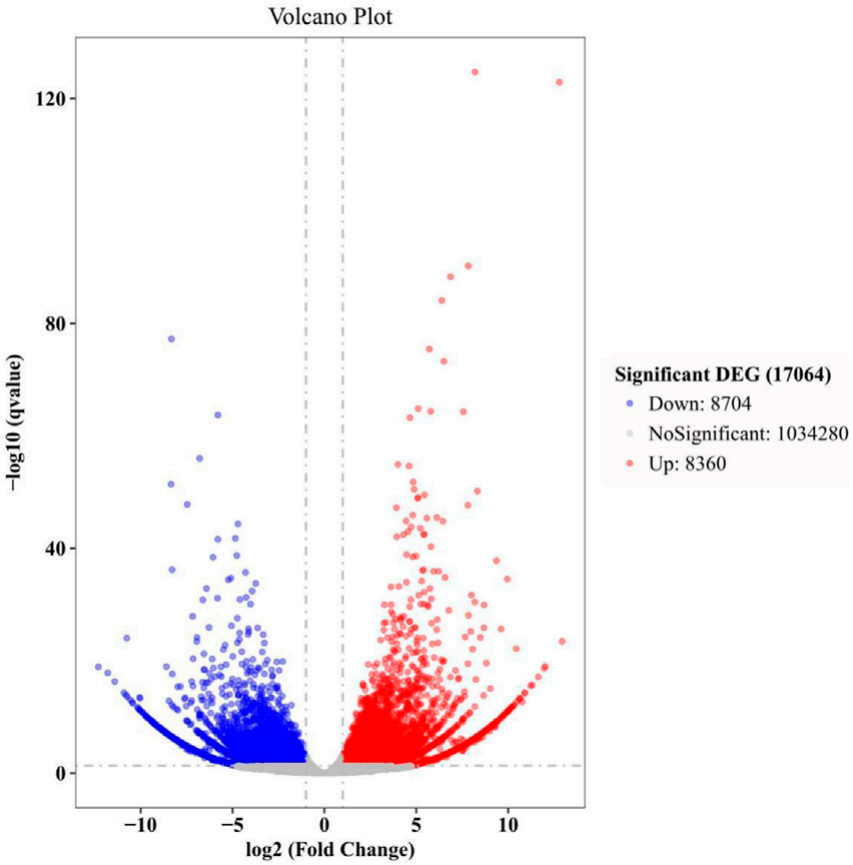
Volcano plot of DEGs in this study.

**FIGURE 4:**
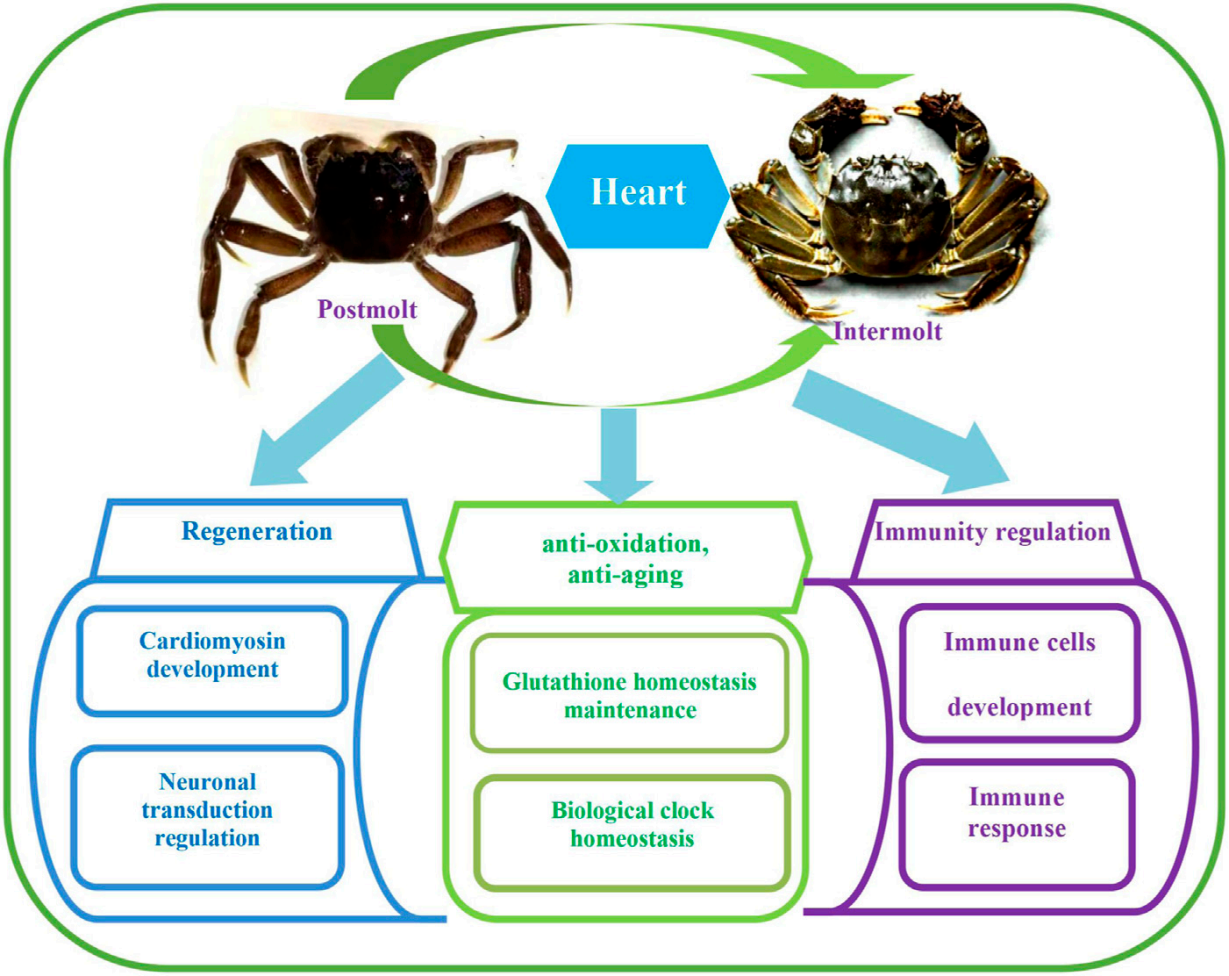
Regulatory network in the heart of *E. sinensis* identified between the post-molt and inter-molt stages.

In this study, there were 17,064 DEGs in total (Figure 3). The top 30 GO terms, top 30 KEGG pathways, and key DEGs can be classified into three categories: regulation of regeneration, regulation of anti-oxidation and anti-aging, and regulation of the immune response. The key functional DEGs identified in this study are listed in Table 3, and all DEGs are shown in Supplementary Material S1. The regulatory network in the heart of *E. sinensis* between the post-molt and inter-molt stages is shown in Figure 4.

**TABLE 3:** Key DEGs in heart transcriptome of *E. sinensis*.

Category	Gene name	Gene definition	log_2_Foldchange	*padj*
Regeneration	*ACTA2*	Actin, aortic smooth muscle	1.537	0.008
	*COX15*	Cytochrome c oxidase assembly protein COX15 homolog	1.133	0.014
	*ARPP21*	cAMP-regulated phosphoprotein 21	1.609	0.021
	*FZD1*	Frizzled-1	2.029	0.031
	*IQGAP1*	Ras GTPase-activating-like protein IQGAP1	4.369	0.000
	*CNN1*	Calponin-1	2.489	0.002
	*FZD7*	Frizzled-7	3.668	0.000
	*BAP60*	Brahma-associated protein of 60 kDa	7.206	0.000
	*WNT2*	Protein Wnt-2	3.262	0.005
	*WNT5B*	Protein Wnt-5b	5.171	0.020
	*NMDAR1*	Glutamate [NMDA] receptor subunit 1	6.389	0.001
	*ATP2B2*	Plasma membrane calcium-transporting ATPase 2	5.779	0.005
	*NTRK2*	BDNF/NT-3 growth factors receptor	6.034	0.003
	*ECR*	Ecdysone receptor	-5.224	0.019
	*UNC-22*	Twitchin	6.974	0.000
	*NOS1AP*	Carboxyl-terminal PDZ ligand of neuronal nitric oxide synthase protein	8.685	0.000
	*BIN1*	Myc box-dependent-interacting protein 1	1.107	0.031
	*TIM*	Protein timeless	2.210	0.045
Antioxidation and anti-aging	*GCLC*	Glutamate--cysteine ligase catalytic subunit	3.617	0.000
	*GGT1*	Glutathione hydrolase 1 proenzyme	1.256	0.000
	*GSTD1*	Glutathione S-transferase 1, isoform C	6.480	0.001
	*DJR-1.1*	Glutathione-independent glyoxalase DJR-1.1	8.008	0.000
	*ERCC2*	General transcription and DNA repair factor IIH helicase subunit XPD	3.737	0.000
	*ALDH3A1*	Aldehyde dehydrogenase, dimeric NADP-preferring	3.037	0.000
	*DAO*	D-amino-acid oxidase	-2.806	0.034
	*RAD3*	The general transcription and DNA repair factor IIH helicase subunit XPD	3.23	0.002
	*ENOX2*	Ecto-NOX disulfide-thiol exchanger 2	8.626	0.000
	*NAMPT*	Nicotinamide phosphoribosyltransferase	2.080	0.005
Immune response regulaiton	*ITGA4*	Integrin alpha-4	4.098	0.000
	*ATG5*	Autophagy protein 5	1.488	0.019
	*KIFAP3*	Kinesin-associated protein 3	4.712	0.047
	*LGMN*	Legumain	2.024	0.000
	*NFYC*	Nuclear transcription factor Y subunit gamma	1.286	0.002
	*PCNA*	Proliferating cell nuclear antigen	2.856	0.000
	*SPON2*	Spondin-2	1.567	0.000
	*CBLB*	E3 ubiquitin-protein ligase CBL-B	6.695	0.000

### Validation of transcriptome data by qPCR

**FIGURE 5:**
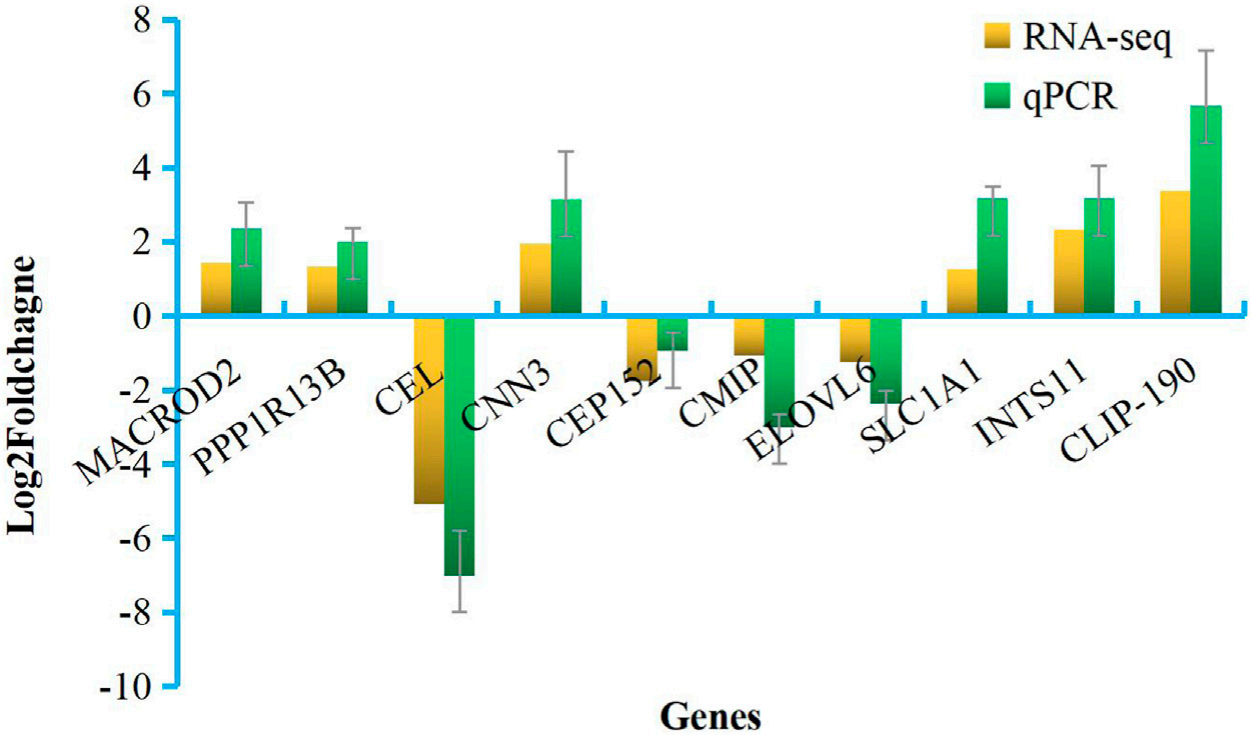
Validation of RNA-seq data by qPCR. *X*-axis, detected gene names; *Y*-axis, the relative expression level was expressed as log2 (fold change) in gene expression.

**FIGURE 6:**
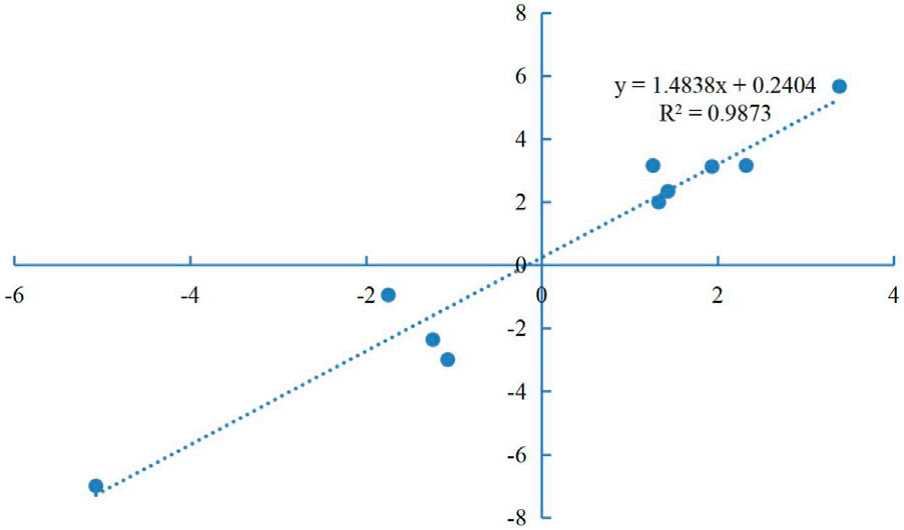
Correlation analysis on the detected DEGs of qPCR.

Primers for 10 of the DEGs identified here are shown in Supplementary Material S2. The relative expression levels of these DEGs as measured by qRT-PCR were consistent with those determined by high-throughput sequencing (Figure 5), indicating the reliability of the transcriptome data. Correlation analysis was shown in Figure 6.

## Discussion

This study identified regulatory pathways and functional genes relevant to tissue regeneration, anti-oxidation, anti-aging and immune regulation that were differentially expressed after *E. sinensis* molting compared to the inter-molt stage.

### Tissue regeneration in the post-molt stage

Several pathways involved in tissue regeneration including the Hippo signaling pathway, Rap1 signaling pathway, thyroid hormone signaling pathway and apoptosis were significantly upregulated at the post-molt stage of *E. sinensis*.

The Hippo signaling pathway is highly conserved among different species and was first discovered in *drosophila*, where it plays an important regulatory role in cell proliferation, differentiation and migration, and organ size control (Fulford et al., 2018; Hong et al., 2018). The Wnt pathway is also highly conserved. Relevant studies have mainly been conducted on vertebrate species and indicate the Wnt signaling pathway plays an important regulatory role in cardiac development. Wnt binds to Frizzled receptor, with both molecules being essential regulators of cardiac development. Inhibition of the Wnt signaling pathway can block cardiac development during the early differentiation of human pluripotent stem cells (Pahnke et al., 2016; Guo et al., 2019; Kaplan et al., 2019). Rap1 is a small GTPase protein with high homology to Ras protein. It acts as a molecular switch and plays an important role in modulating cell movement and the formation of cellular connections. Rap1 can participate in the regulation of tight connections and in the formation of adhesion connections between epithelial cells and endothelial cells, thus affecting the integrity of barrier functions (Yang et al., 2021). Apoptosis is the spontaneous and orderly death of cells required to maintain internal environmental homeostasis. It plays an important regulatory role in the evolution of internal homeostasis and in the development of many organ systems. The release of cytochrome C from mitochondria is a key step in apoptosis. Caspase can act on several enzymes related to cytoskeleton regulation and hence alter the cell structure (Klemm et al., 2021). Damaged cells undergo apoptosis to clear out irreparable cells, thereby initiating tissue regeneration (Onishchenko et al., 2021).

In the present study we found differential expression of hippo, Rap1 and apoptosis pathway genes, as well as some regulatory genes relevant to cardiac development, heart rate regulation and calcium signal transduction in the neuronal system. The WNT-5B developmental protein together with frizzled receptors play a modulatory role in tissue morphogenesis (Ghosh et al., 2008; Yu et al., 2010; Sunkara et al., 2021). Studies on *Xenopus* have shown that FZD7 is required for heart development (Abu-Elmagd et al., 2017). In the present study, WNT-5B, FZD1 and FZD7 were all up-regulated after molting, suggesting they play a synergistic regulatory role in the cardiac development of *E. sinensis* during the post-molt period. COX15 is essential for the synthesis of heme A and plays a regulatory function in the blood circulatory system (De Oliveira et al., 2021). Myocytes invaginate to form T-tubes and prevent the negative effects of rapid changes in extracellular fluid induced by calcium. BIN1 plays a regulatory role in T-tube formation (Draeger et al., 2017). BIN1 also modulates calcium flow and cardiac myocyte movement. The function of UNC-22 is to modulate muscle contraction and relaxation (Matsunaga et al., 2015). CNN1 plays a regulatory role in muscle contraction via binding to actin and calmodulin (Feng et al., 2019). ARPP21 has a negative regulatory role for calmodulin-dependent enzymes. In the present study, upregulation of ARPP21 expression may help to maintain the homeostasis of cardiac myocyte contraction (Chen et al., 2018). IQGAP1 plays a modulatory role in cytoskeleton assembly of actin (Cheung et al., 2013). ATP2B2 has an active regulatory role in calcium homeostasis in neuronal systems (Martin-De-Saavedra et al., 2022). Glutamate is an important excitatory neurotransmitter that together with its receptor has a regulatory role in autonomy, conductivity and self-discipline (Hearn et al., 2022). Research on cultured rat myocardial cells indicate that glutamate can increase the concentration of calcium, thus increasing the contraction rate of cardiac myocytes (Hasan and Nabika, 2021). GRIN1 plays a positive role in regulating myocardial contraction. BAP60 participates in the regulation of neurogenesis (Koe et al., 2014). NTRK2 plays a regulatory role in the development and maturation of central and peripheral nervous systems and synaptic plasticity (Pattwell et al., 2021).

The heart of crustaceans is neurogenic. Cardiac ganglion in the heart modulates cardiac signal transduction and initiates and regulates cardiac myocyte contraction (Garcia-Crescioni et al., 2010). In the present study, some of the regulatory DEGs involved in heart development after molting were related to neuronal signal transduction, myocardial movement, heart development and apoptosis.

### Anti-oxidation and anti-aging at the post-molt stage

Aging is a complex natural phenomenon that manifests as a decline in physiological function, weakened resistance to the environment, slower metabolism and slower response to stress. The free radical theory is one of the most convincing modern theories to explain the aging mechanism (Cai et al., 2020b). High concentrations of free radicals and their derivatives in tissues have harmful effects on biological macromolecules and can accelerate the aging process. The scavenging of free radicals and subsequent prevention of lipid peroxidation can improve the anti-oxidation capacity, thus causing a delay in aging (Fernandez-Marcos and Nobrega-PereiraNADPH, 2016; Hajam et al., 2022).

Glutathione (GSH) is an important non-enzymatic antioxidant and efficient nucleophile. GSH reacts with electrophiles to remove harmful metabolites such as free radicals. Its concentration is an important indicator of the antioxidant capacity of the body (Lopez-Navarro et al., 2020). In the present study, some of the DEGs were related to the “glutathione metabolic process/glutathione transferase activity/oxidation-reduction process/anti-aging”. GCLC is an essential component for GSH biosynthesis (Fu et al., 2020). GGT1 also plays an active role in the regulation of cysteine homeostasis and glutathione homeostasis (West et al., 2013). DJR-1.1 participates in the detoxification of endogenous glyoxal and in the protection of cell death induced by glyoxal (Hasim et al., 2014). The NMDA receptor is an excitatory neurotransmitter with a critical role in regulating synaptic plasticity, memory, etc. DAO can degrade D–serine, thereby inhibiting NMDA (Lin et al., 2017). In the present study, downregulation of DAO had a positive role in the delay of aging. ALDH3A1 inhibits lipid peroxidation (Black et al., 2012). Upregulation of ALDH3A1 observed in this study enhances the removal of toxic substances and strengthens the anti-aging capability. The accumulation of protein synthesis errors is also an important contributor to aging. Upregulation of RAD3 is conducive to the fidelity of DNA replication, which ultimately benefits protein synthesis (Yeo et al., 2020). Thus, RAD3 can delay the aging process. Many physiological activities have circadian rhythms and the biological clock is closely related to aging. Dysfunction of the biological clock seriously affects the physiological and behavioral rhythms of organisms, leading to endocrine disorders and the acceleration of aging (Radman, 2012). ENOX2 has a positive regulatory role in the organism’s biological clock (Morre and Morre, 2008). NAMPT acts to maintain biological clock homeostasis and thus prevent aging (Khaidizar et al., 2021).

Surprisingly, some of the pathways and regulatory genes involved in anti-oxidation (regulation of GSH homeostasis, inhibition of lipid peroxidation), anti-aging (regulation of biological clock homeostasis) were found in this study to be upregulated during the post-molt stage of *E. sinensis*. In contrast to vertebrates, the heart of *E. sinensis* has strong regenerative ability. Regeneration is the opposite process to aging and hence this study could provide a theoretical framework for research into the anti-aging molecular mechanism in vertebrates.

### Immune regulation during the post-molt stage

The migration of white blood cells from the blood to tissues (leukocyte transendothelial migration) is essential for immune surveillance and inflammation. Inflammatory cells migrate from peripheral blood vessels to inflammatory sites under the stimulation of inflammatory factors, resulting in an immune response (Van Steen et al., 2021). Antigen processing and presentation is the process by which antigen molecules are captured by antigen presenting cells, digested into peptides and then combined with MHC molecules to form complexes that are presented at the cell surface and recognized by immunoactive cells (Gannage et al., 2019).

In this study, some regulatory genes related to immune cell development and immune response regulation were differentially expressed after molting. CBLB has a negative regulatory role with regard to lymphocyte receptors (Nanjappa et al., 2020). The down-regulation of CBLB observed here may help to maintain immune response homeostasis.

During their lifetime, cells face a variety of endogenous and exogenous stresses, including protein misfolding, organelle damage, nutrient deficiency and pathogen invasion. Autophagy is an important way for cells to respond to these stresses. The substances to be removed are wrapped and then transported to lysosomes for degradation (Rakesh et al., 2022; Zhou et al., 2022). ATG5 is an essential component in the formation of autophagy vesicles and plays a key regulatory role in many aspects of lymphocyte development and proliferation (Kim et al., 2020). Upregulation of ATG5 has a positive regulatory role in immune system development and in the immune response. ITGA4 triggers the aggregation of homogenous leukocyte lines onto activated endothelial cells and is involved in T-cell interactions with target cells. In the present study, ITGA4 contributed to enhancement of the immune response. Asparagine is an essential component for the assembly of MHC class I molecules (Fu et al., 2022). LGMN has strict specificity for the hydrolysis of asparagine bonds and participates in the processing of MHC Class II antigen-presenting proteins in the lysosomal/endosomal system. Research conducted in vertebrates has shown that LGMN can promote cardiac repair (Jia et al., 2022). In the present study, LGMN was beneficial for antigen processing and presentation during the immune response and may also play a positive role in tissue repair and regeneration after *E. sinensis* molting. SPON2 acts as an opsonin for macrophages. It binds directly to bacteria and is critical for initiating innate immune responses (Zhou et al., 2021). SPON2 may have a positive regulatory role by enhancing resistance to pathogenic microorganisms during the post-molt period of *E. sinensis*.

## Conclusion

In this study, comparative transcriptome analysis was carried out on the heart of E. sinensis at the post-molt and inter-molt stages. The results showed significant differential expression of many regulatory pathways and genes involved in regeneration, antioxidation, anti-aging and the immune response. Aside from cardiac development and with regard to the regulation of regeneration, these DEGs were relevant to myocardial movement and to neuronal signal transduction. With regard to antioxidation and anti-aging, the DEGs were involved with regulation on GSH homeostasis and biological rhythms. With regard to the immune response, the DEGs were involved in the regulation of immune cell development and the immune response. This study provides a theoretical background for further research into regulatory mechanisms in *E. sinensis* and other economically valuable crustaceans (eg. procambarus clarkii) and the crustacean breeding industry in general. In contrast to vertebrates, the heart of *E. sinensis* has strong regenerative potential. This study may provide a theoretical framework for further research into the regulatory mechanisms of organ regeneration and anti-aging in vertebrates.

## Data Availability

The raw data supporting the conclusion of this article will be made available by the authors, without undue reservation.

## Abbreviations

E. sinensis, Eriocheir sinensis; DEGs, differentially expressed genes; VATB, V-ATPase subunit B; TGFBR1, transforming growth factor-beta type I receptor; MP, post-molt stage; MI, inter-molt stage; Nr, Non-redundant protein; Nt, Non-redundant nucleotides; KOG, Clusters of orthologous groups for eukaryotic complete genomes; KEGG, Kyoto Encyclopedia of Genes and Genomes; GO, Gene Ontology; qPCR, Quantitative real-time polymerase chain reaction; BP, biological process; MF, molecular function; CC, cellular component; GSH, Glutathione.
