# Supplementary material for: Exploration of the regulatory mechanisms of regeneration, anti-oxidation, anti-aging and the immune response at the post-molt stage of Eriocheir sinensis
Source: Front Physiol. 2022 Sep 21;13:948511. doi: 10.3389/fphys.2022.948511 (PMC9552667; doi:10.3389/fphys.2022.948511)
Supplement: Supplementary file 1 [file DataSheet1.zip › Supplementary files/Supplementary file 2.doc]

| Table S2. The primers of the genes used for Real-time RT-PCR validation | | | |
| --- | --- | --- | --- |
| Number | Gene name | Definition | Sequence (5’→3’) |
| 1 | MACROD2 | ADP-ribose glycohydrolase MACROD2 | F:TGAAAGAAGATTCAGATGAGAACGG |
|  |  |  | R:ACCGCCTTTTGTAATATTTTCATCC |
| 2 | PPP1R1 | Apoptosis-stimulating of p53 protein 1 | F:CTGGAAGGAGAGTTCGATCTGG |
|  |  |  | R:CTGCACCCCATCCATCACTAT |
| 3 | CEL | Bile salt-activated lipase | F:AAAGGTGGCTGAGAAGGTGG |
|  |  |  | R:GTCAGCGGGGATGAAGTCTC |
| 4 | CNN3 | Calponin-3 | F:GCCTGAGCATTGGTGCAAAC |
|  |  |  | R:TGTGGCTTCATGCCATAGACT |
| 5 | CEP152 | Centrosomal protein of 152 kDa | F:AGAGTGTAAAAGCCAGCCCC |
|  |  |  | R:CCTTGGCAAAATGTCTAGGTGT |
| 6 | CMIP | C-Maf-inducing protein | F:TCGTCTCGAAACTGCTCTCAGGAG |
|  |  |  | R:GACCACAGGCGTGAAAACTTC |
| 7 | ELOVL6 | Elongation of very long chain fatty acids protein 6 | F:TCGCTCGATCTCAGCATGTC |
|  |  |  | R:CCGCATCACCGTTTTTCAAGT |
| 8 | SLC1A1 | Excitatory amino acid transporter 3 | F:GAAGCAGCTACAAGCAAGC |
|  |  |  | R:AGCGTATCTGCGGTAAGGAC |
| 9 | INTS11 | Integrator complex subunit 11 | F:CGAGGCCAACTTCTTCACCT |
|  |  |  | R:CACCCGTGTAGACCACAGAC |
| 10 | CLIP-190 | Restin homolog | F:AGCTCTATGCTTCGCAGTCC |
|  |  |  | R:GTTCCATCGTTTTTGCCGCT |
| 11 | β-actin | β-actin | F: GCAACACGCAGCTCGTTGTAG |
|  |  |  | R: CAGGCATCAGGGTGTGATGG |
